# Caco-2 Cell Co-Culture Alters the Molecular Size of Igl1 and Its Extracellular Fragments in *Entamoeba histolytica*

**DOI:** 10.3390/pathogens15060633

**Published:** 2026-06-15

**Authors:** Kentaro Kato, Mizuki Kudo, Hideaki Unno, Tomomitsu Hatakeyama, Hiroshi Tachibana

**Affiliations:** 1 Department of Eco-Epidemiology, Institute of Tropical Medicine (NEKKEN), Nagasaki University, 1-12-4 Sakamoto, Nagasaki 852-8523, Nagasaki, Japan; 2 School of Tropical Medicine and Global Health, Nagasaki University, 1-12-4 Sakamoto, Nagasaki 852-8523, Nagasaki, Japan; 3 Graduate School of Engineering, Nagasaki University, 1-14 Bunkyo-machi, Nagasaki 852-8521, Nagasaki, Japan; mizuki.dori@docomo.ne.jp (M.K.); tmhata@gmail.com (T.H.); 4 Graduate School of Integrated Science and Technology, Nagasaki University, 1-14 Bunkyo-machi, Nagasaki 852-8521, Nagasaki, Japan; unno@nagasaki-u.ac.jp; 5 Organization for Marine Science and Technology, Nagasaki University, 1-14 Bunkyo-machi, Nagasaki 852-8521, Nagasaki, Japan; 6 Department of Parasitology, School of Medicine, Tokai University, 143 Shimokasuya, Isehara 259-1193, Kanagawa, Japan; htachitachi55@gmail.com

**Keywords:** *Entamoeba histolytica*, Igl1, Gal/GalNAc lectin, cysteine protease, protein fragmentation

## Abstract

The galactose/*N*-acetyl-D-galactosamine (Gal/GalNAc)-inhibitable lectin of *Entamoeba histolytica* plays essential roles in host cell adhesion and cytotoxicity. The intermediate subunit lectin-1 (Igl1) contributes to these functions, but its molecular state under different environmental conditions remains unclear. In this study, we found that Igl1 is present as multiple fragments in the culture supernatant of trophozoites, whereas a single major species corresponding to intact Igl1 was detected in cell lysates. Notably, the molecular sizes of both intact Igl1 and its extracellular fragments differed depending on culture conditions, with larger apparent sizes observed under co-culture with Caco-2 cells. These differences were not explained by changes in transcript levels, protein folding, or N-terminal truncation. Fragmentation of Igl1 was suppressed by a cysteine protease inhibitor, indicating extracellular generation. These findings demonstrate that host-cell-associated conditions alter the molecular size of Igl1 and that extracellular protease-dependent processing generates multiple Igl1 fragments, providing new insights into the regulation of this key virulence factor. The presence of extracellular fragments further suggests a potential contribution to host tissue damage during amoebiasis.

## 1. Introduction

*Entamoeba histolytica* (*E. histolytica*) causes amoebiasis, with an estimated 50 million cases of dysentery, colitis, and extraintestinal abscesses annually, resulting in approximately 55,000 deaths worldwide [1]. Galactose (Gal)- and *N*-acetyl-D-galactosamine (GalNAc)-inhibitable lectins are essential for the adherence of *E. histolytica* trophozoites to colonic mucins and host cells [2]. These lectins consist of a heteromultimer composed of a 170-kDa transmembrane heavy subunit (Hgl) and a 30/35 kDa glycosylphosphatidylinositol (GPI)-anchored light subunit (Lgl). In addition, the 150 kDa GPI-anchored intermediate subunit (Igl) is non-covalently associated with the Hgl/Lgl complex in distinct membrane domains and contributes to host cell adhesion [3,4,5]. Two isoforms of Igl, Igl1 and Igl2, have been identified; both contain multiple cysteine residues and CXXC motifs but differ in expression levels and subcellular localization in trophozoites [6,7]. We previously demonstrated that both Igl proteins exhibit hemolytic activity, and that Igl1 additionally possesses cytotoxic activity [8,9]. These activities are associated with multiple regions located in the C-terminal portion of Igl1, which is positioned close to the plasma membrane of trophozoites [10]. Despite the importance of Igl1 in parasite virulence, its molecular state under different environmental conditions remains poorly understood. In particular, it is unclear whether Igl1 undergoes structural or proteolytic changes in response to host-associated conditions.

In this study, we showed that Igl1 exists as multiple fragments in the culture supernatant of *E. histolytica* trophozoites and that both intact Igl1 and its extracellular fragments exhibit differences in molecular size depending on culture conditions.

## 2. Materials and Methods

### 2.1. Cell Culture

*Entamoeba histolytica* (HM1:IMSS cl6) trophozoites were maintained in TYI-S-33 medium, and human colon adenocarcinoma Caco-2 cells (HTB-37, ATCC, Manassas, VA, USA) were maintained in MEM basic medium (Gibco, Grand Island, NY, USA) supplemented with Earle’s salts, L-glutamine, and 20% fetal bovine serum. For this study, sub-confluent cultures of Caco-2 cells in a 25 cm^2^ culture flask (163371, Thermo Scientific, Roskilde, Denmark) were harvested and suspended in MEM medium. The Caco-2 cells were seeded at 2 × 10^5^ cells/1.2 mL/well in 48-well plates (150687, Thermo Scientific, Roskilde, Denmark) and grown until the cells reached approximately 80% confluence. When the Caco-2 cells reached confluence, the MEM medium was removed from each well, 1.2 mL of TYI-S-33 medium containing *E. histolytica* (0.5 × 10^5^ trophozoites/well) was added to the wells, and the plates were incubated for an additional 24 h at 37 °C under 5% CO_2_. Samples from single cultures of Caco-2 cells were prepared as described above, except that 1.2 mL of TYI-S-33 medium without trophozoites was added to the wells and incubated for an additional 24 h at 37 °C. The resulting culture supernatant (hereafter referred to as Caco-2-conditioned TYI-S-33 medium) was used for subsequent experiments. Single cultures of *E. histolytica* trophozoites were also prepared by spreading 0.5 × 10^5^ trophozoites in each well of the plate and incubated for 24 h at 37 °C under 5% CO_2_. Culture supernatants from the 24 h culture were collected.

For cell lysate preparation, *E. histolytica* trophozoites were lysed with 200 μL of 0.2% Triton X-100 in phosphate-buffered saline (PBS, pH 7.4) per well. *E. histolytica* trophozoite lysates from co-culture preparations were obtained after removal of detached dead Caco-2 cells from the wells by brief washing with PBS. Cell images were captured under an ECLIPSE Ts2 microscope (Nikon, Tokyo, Japan) equipped with a WRAYCAM VEX830 camera (WRAYMER, Osaka, Japan). The images were analyzed using MicroStudio software (ver. x64, 1.7.20131.20220108, WRAYMER, Osaka, Japan).

trans-Epoxysuccinyl-L-leucylamido-(4-guanidino)butane (E-64, Peptide Institute, Inc., Osaka, Japan), a cysteine protease inhibitor, was purchased. *E. histolytica* trophozoites were seeded at 1× 10^5^ trophozoites/well/1.2 mL medium in a 48-well plate and grown for 24 h at 37 °C. Then, the culture supernatants were removed and replaced with either medium with 100 μM E-64 or with the same amount of H_2_O. Culture supernatants from the additional 24 h culture were collected, and the number of *E. histolytica* trophozoites was counted at seeding (0 h), medium replacement (24 h), and medium collection (48 h).

### 2.2. Anti-Igl1 Monoclonal Antibodies

Purified humanized anti-Igl1 monoclonal antibodies, XEhI-20 (IgG2) and XEhI-H2 (IgM), were prepared as previously described [11]. XEhI-20 recognizes a conformational epitope of Igl1 and XEhI-H2 recognizes the middle region to the C-terminus of Igl1 [8,9].

### 2.3. Immunoprecipitation and Purification of Igl1 Fragments

Anti-Igl1 monoclonal antibody (XEhI-20) was covalently coupled to Dynabeads M-270 Epoxy following the protocol supplied with the Dynabeads™ Antibody Coupling Kit (14311D, Thermo Fisher, Vilnius, Lithuania) at 10 μg Ab/mg beads. The culture supernatants and cell lysates of *E. histolytica* trophozoites were prepared as described above. One milliliter of culture supernatant or 50 μL of cell lysate was mixed with 1 mg of antibody-immobilized beads, and the samples were incubated at 4 °C overnight with rotation. As a control, TYI-S-33 medium incubated with Caco-2 cells under the same conditions (Caco-2-conditioned TYI-S-33 medium) was used. The beads were washed three times with 1 mL of Tris-buffered saline with 0.1% Tween-20 (TBST, pH7.4). The bound proteins were eluted with 100 μL of 50 mM glycine–HCl (pH 2.8) and neutralized by adding 10 μL of 1 M Tris-HCl (PH 8.5). The eluted proteins were separated by SDS-PAGE and detected as described below. In some experiments, hundred-fold concentrated samples of eluted proteins were prepared using Amicon Ultra centrifugal filter units (MWCO 10 kDa; Millipore, Carrigtwohill, Ireland), and IgGs in the samples from culture media were removed by treating the samples with protein G-conjugated magnetic beads (0433, Ademtech, Pessac, France) at 4 °C overnight with rotation.

### 2.4. SDS-PAGE, Coomassie Brilliant Blue Staining and Western Blot Analyses

Samples were loaded onto 8% Bis-Tris gels (Thermo Fisher Scientific, Carlsbad, CA, USA) and electrophoresed with MOPS running buffer at 200 V for 35–40 min under either reducing or non-reducing conditions. For reducing conditions, the samples were treated with NuPAGE™ Sample Reducing Agent (Thermo Fisher Scientific, Carlsbad, CA, USA) prior to electrophoresis. The proteins in the samples were visualized using Coomassie Brilliant Blue (CBB) staining by treating the gels with SimplyBlue Safe Stain solution (Thermo Fisher Scientific, Carlsbad, CA, USA).

For Western blot analysis, the proteins were transferred to nitrocellulose membranes for 7 min using an iBlot2 dry blotting system (Thermo Fisher Scientific, Kiryat Shmona, Israel). The membranes were blocked in 3% BSA/PBS, followed by incubation with primary antibodies (1:1000 dilution of 1 mg/mL anti-Igl1 antibody in 3% BSA/PBS) at 4 °C overnight. After washing three times with TBST, the membranes were incubated with alkaline phosphatase (AP) goat anti-human IgG (H+L) (ab7154, Abcam, Cambridge, UK) for 1 h at room temperature. The membranes were washed three times in TBST and once in H_2_O. The proteins bound to the antibodies were visualized in 1-step NBT/BCIP substrate solution (34042, Thermo Fisher, Rockford, IL, USA). Recombinant Igl1 expressed in *Escherichia coli* was prepared as previously described [8] and used as a positive control for immunoblotting. Band signals on the membranes were obtained using ImageJ software ver. 1.54g (NIH, Bethesda, MD, USA) [12].

### 2.5. Real-Time PCR Analysis

Real-time PCR was performed as previously described with slight modifications [9,13]. Briefly, the total RNA of *E. histolytica* in each preparation was isolated using an RNeasy Mini Kit (Qiagen). The RNAs were treated with DNaseI (Takara, Shiga, Japan), and reaction mixtures for quantitative real-time PCR were prepared using specific primers [13] and a QuantiNova SYBR Green RT-PCR kit (208152, Qiagen, Hilden, Germany). Forty cycles of amplification, with recording of fluorescence intensity in each cycle, were performed using an Applied Biosystems 7500 Real-Time PCR System (ABI, Foster City, CA, USA). Expression levels of *Igl1* genes were analyzed with 7500 Software v2.3 (ABI, Foster City, CA, USA) using the comparative Ct method with actin as an internal standard. The experiments were repeated four times, including the culture and RNA isolation.

### 2.6. N-Terminus Peptide Sequencing

Twenty microliters of 100-fold concentrated samples were loaded onto 15% gels, and SDS-PAGE was conducted. Proteins in the samples were blotted on PVDF membranes. The membranes were stained with 0.1% (*w*/*v*) Ponceau S (Nacalai Tesque, Kyoto, Japan) in a 5% (*w*/*v*) acetic acid solution. Stained bands were excised from the membranes and applied to the protein sequencer PPSQ-21 (Shimadzu, Kyoto, Japan).

### 2.7. Statistical Analysis

Statistical analysis was performed using Student’s t-test or one-way ANOVA followed by the Tukey–Kramer multiple comparison test, as appropriate. Four independent biological samples were analyzed for mRNA expression analysis, and three independent biological replicates were analyzed for the E-64 treatment experiments. The exact *p* value for mRNA expression is indicated in the relevant figure. *p* < 0.05 and *p* < 0.01 were considered statistically significant.

## 3. Results

### 3.1. Igl1 Fragments Were Present in the E. histolytica Culture Supernatant

Igl1 exhibits both hemolytic and cytotoxic activities at multiple sites in the C-terminus close to the GPI-anchored region of the protein [8,10]. To explore the mechanism underlying this activity, we hypothesized that the C-terminal region of Igl1 may be exposed through proteolytic processing. This could occur either by cleavage that retains the region on the parasite surface or by release of fragments into the extracellular environment. Based on this hypothesis, we examined whether Igl1 fragments are present in the culture supernatant of *E. histolytica* trophozoites. To determine whether Igl1 was present in culture supernatants, we examined culture supernatants of trophozoites (Supplemental Figure S1A). Igl1 was immunoprecipitated from the culture supernatants of *E. histolytica* trophozoites using an anti-Igl1 monoclonal antibody (XEhI-20), which recognizes a conformational epitope, and then subjected to SDS-PAGE, transferred to membranes, and detected with the monoclonal antibody.

There were at least three molecular species of Igl1 with sizes of approximately 120 kDa (band 1), 90 kDa (band 2), and 60 kDa (band 3) in the culture supernatant of *E. histolytica* trophozoites. The bands with asterisks are IgG-derived proteins in the culture medium that can be easily depleted by treating the sample with protein G beads (Supplemental Figure S1A).

### 3.2. The Molecular Size of Igl1 Fragments Varies Under Different Culture Conditions of E. histolytica Trophozoites

**Figure 1:**
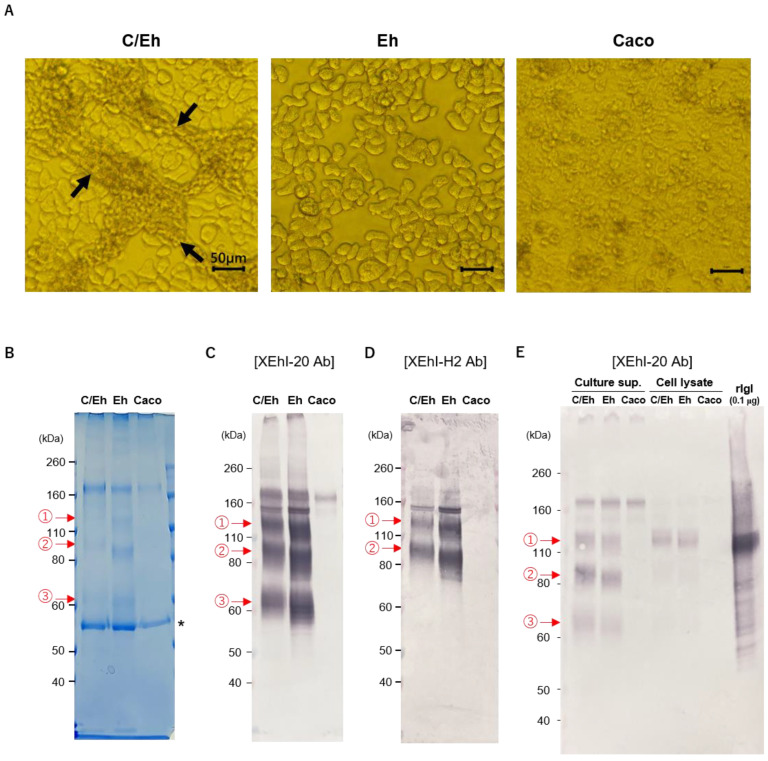
Three molecular species of Igl1 in culture supernatants and cell lysates of *Entamoeba histolytica* in different culture conditions. (**A**) Cell images after 24 h of Caco-2 and *E. histolytica* co-culture (C/Eh), *E. histolytica* single culture (Eh) or Caco-2 single culture (Caco). Arrows indicate detached Caco-2 cell sheets generated following destruction of the Caco-2 monolayer by *E. histolytica*. Bars indicate 50 μm. (**B**) CBB-stained gel images of purified Igl1 species from the culture supernatants in each culture condition. Red arrows indicate the bands of Igl1 species. An asterisk indicates bovine serum albumin. (**C**) Western blot analysis of Igl1 species from culture supernatants with XEhI-20 antibody. (**D**) Western blot analysis of Igl1 species from culture supernatants with XEhI-H2 antibody. (**E**) Western blot analysis of Igl1 species in non-concentrated Igl1 samples from culture supernatants and cell lysates with XEhI-20 antibody. rIgl: recombinant Igl (120 kDa). The samples were run in 8% Bis-Tris gels.

Since Igl1 is cytotoxic, we expected that the expression levels of Igl1 molecular species would vary in different culture conditions of *E. histolytica* trophozoites. For this experiment, trophozoites were co-cultured with Caco-2 cells (C/Eh) or cultured alone (Eh). The culture supernatant from the Caco-2 single culture (Caco) was used as a negative control (Figure 1). After 24 h of co-cultivation with *E. histolytica* and Caco-2 cells, dead Caco-2 cells detached from the culture plate and flowed over the *E. histolytica* monolayer (Figure 1A, arrows). Caco-2 cells were confirmed as dead cells by 0.4% trypan blue staining (Supplemental Figure S1B). In contrast, Caco-2 cells in single culture conditions had a cobblestone appearance, as shown in Figure 1A (Caco). Igl1 in the culture supernatants from each culture condition was purified using XEhI-20 mAb-conjugated beads. The protein expression levels of Igl1 molecular species in the C/Eh and Eh culture supernatants did not differ (Figure 1B). This was also confirmed by Western blotting using XEhI-20 mAb (Figure 1C) and C-terminus-recognizing mAb, XEhI-H2 (Figure 1D). These results indicate that the 60 kDa Igl1 species (band 3) lacked the C-terminal region of the protein. The Igl1 molecular species around 120 kDa was the major species in *E. histolytica* trophozoite lysates, and the sizes were slightly larger than the 120 kDa fragments in the culture supernatants (Figure 1E), indicating that Igl1 fragments in the culture supernatants were generated by proteolytic cleavage of Igl1 on the cellular membranes of the trophozoites. Igl1 molecular species from C/Eh culture supernatant and cell lysates had slightly larger molecular sizes than those from Eh culture supernatant and lysates (Figure 1B–E).

### 3.3. The Expression Levels of Igl1 mRNA Did Not Differ Between Samples

**Figure 2:**
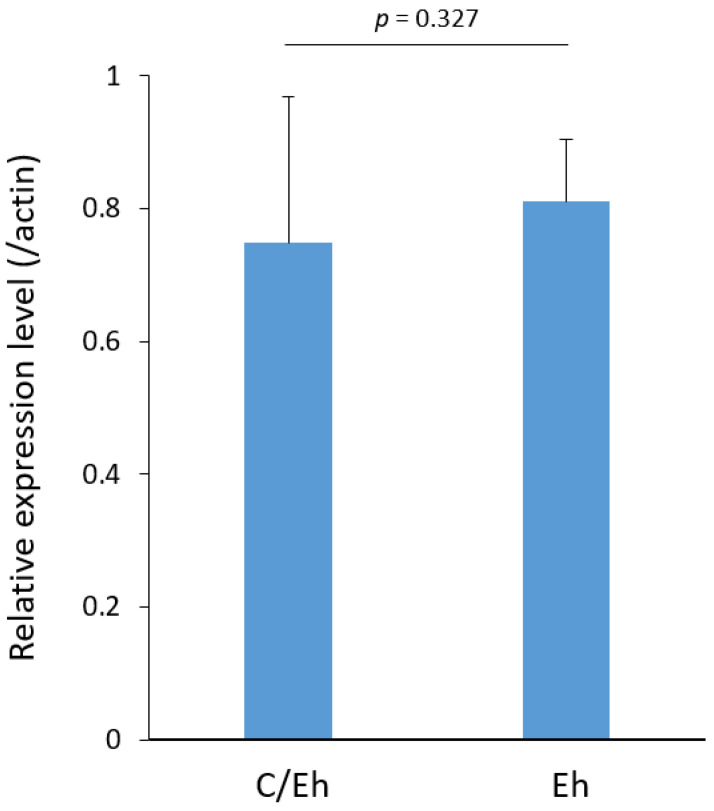
Relative expression of *Igl1* in *Entamoeba histolytica* in different culture conditions. *Igl1* expressions in *E. histolytica* cultured alone (Eh) or with Caco-2 cells (C/Eh) are compared. The results are shown as the average of relative *Igl1* expression levels against actin levels from four independent samples, together with standard deviation values. Student’s t-test was applied for the statistical analysis. The exact *p* value is indicated in the figure.

As shown in Figure 1B–E, no obvious difference in the band intensities of Igl1 species was observed between the culture conditions. To further examine whether the observed molecular size alterations were associated with changes in gene expression, we compared the mRNA levels of *Igl1* under these culture conditions. There was no significant difference in *Igl1* mRNA expression between the two culture conditions (Figure 2).

### 3.4. Protein Folding and N-Terminal Sequences of the Igl1 Species Did Not Differ Under Different Culture Conditions

**Figure 3:**
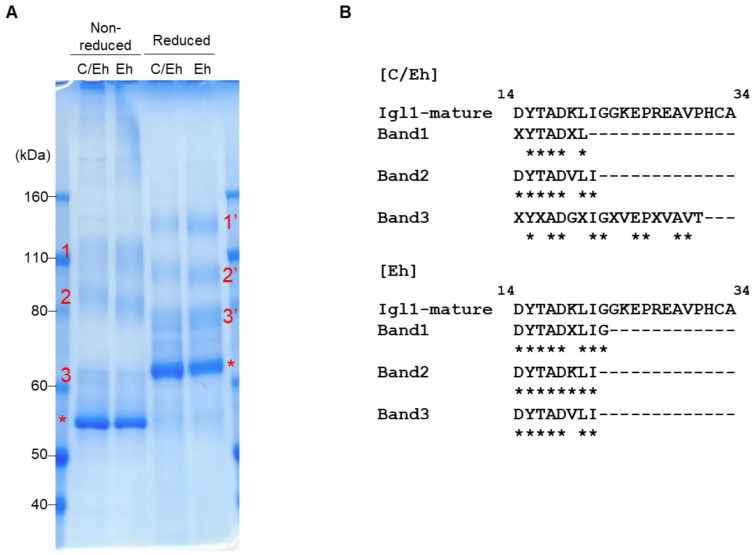
Protein folding and N-terminal sequences of Igl1 species in different culture conditions. (**A**) CBB staining images of non-reduced and reduced Igl1 species purified from culture supernatants in different culture conditions. C/Eh: Caco-2 and *E. histolytica* (co-cultured). Eh: *E. histolytica* (single cultured). (**B**) N-terminal sequences of Igl1 species are shown. Those sequences are compared with the partial N-terminal sequence of the Igl1 mature protein (aa1-13 is a signal sequence). Asterisks indicate the amino acids matched with the reference sequence. All the fragments retained N-terminal amino acids.

It is possible that folding and/or N-terminus cleavage of the Igl1 species affected their mobilities in the gels because Igl1 is a cysteine-rich protein. However, these possibilities were considered unlikely because the molecular size differences were not completely abolished by reduction (Figure 3A), and the N-terminal sequences of the fragments remained intact, as confirmed by N-terminal peptide sequencing (Figure 3B).

### 3.5. E-64 Cysteine Protease Inhibitor Suppressed Igl1 Fragmentation in E. histolytica Culture Media

**Figure 4:**
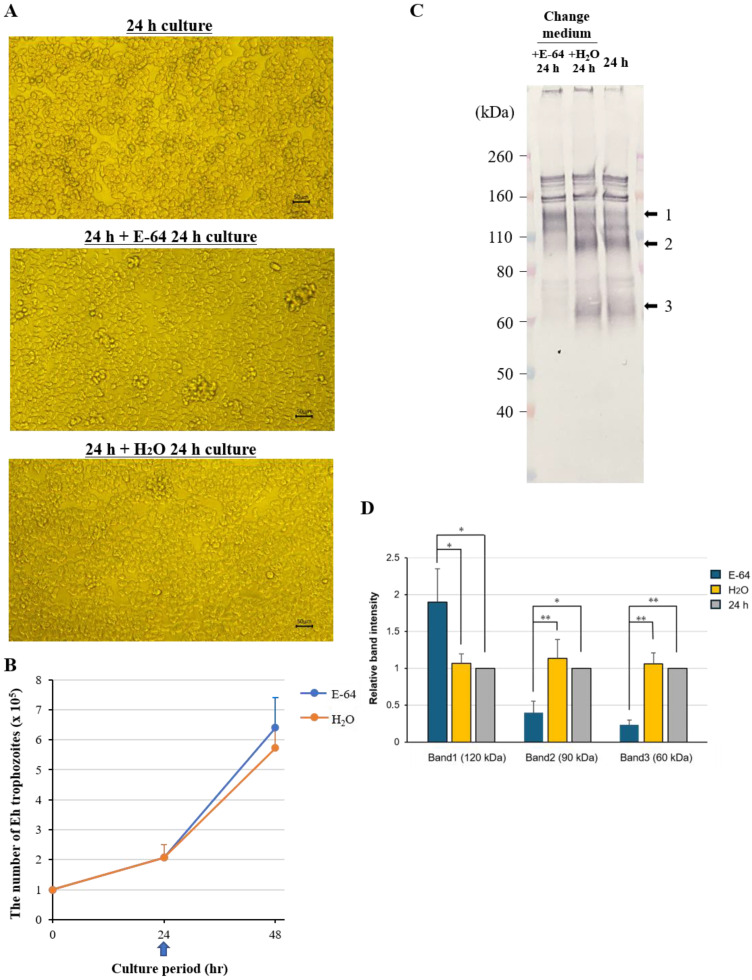
Involvement of cysteine proteases in Igl1 fragmentation. (**A**) Cell images of *E. histolytica* trophozoites in different culture conditions. Bars indicate 50 μm. (**B**) The number of *E. histolytica* trophozoites after replacing the medium with 100 μM E-64-supplemented or control medium. The medium was replaced at 24 h after cultivation, indicated by a blue arrow. (**C**) Western blotting result of Igl1 fragments in culture supernatants from different culture conditions. Three fragments of Igl1 (band numbers 1–3) in culture supernatants were detected by Western blot analysis with XEhI-20 antibody. (**D**) Quantification of the band intensities of the 120 kDa, 90 kDa, and 60 kDa Igl1 fragments shown in panel C. Band intensities were quantified using ImageJ and expressed as relative band intensity. Data are presented as the mean + SD of three independent biological replicates. Statistical comparisons were performed using one-way ANOVA followed by the Tukey–Kramer multiple comparison test. * *p* < 0.05, ** *p* < 0.01.

*E. histolytica* has at least 50 cysteine proteases, of which EhCP1, EhCP2, and EhCP5 account for approximately 90% of the protease activity in the parasite [14]. To examine whether cysteine proteases were involved in the Igl1 fragmentation, *E. histolytica* trophozoites were cultured with a cysteine protease inhibitor, E-64 (Figure 4). Morphological images (Figure 4A) and the growth (Figure 4B) of the trophozoites did not differ among the experimental groups. In contrast, E-64 treatment altered the pattern of extracellular Igl1 fragments, resulting in an increased intensity of the approximately 120 kDa fragment and decreased intensities of the approximately 90 kDa and 60 kDa fragments (Figure 4C). Quantitative analysis of three independent experiments confirmed these changes to be statistically significant (Figure 4D), indicating that cysteine protease activity is involved in Igl1 fragmentation.

## 4. Discussion

In the present study, we demonstrated that the molecular sizes of both intact Igl1 and its extracellular fragments differ between *E. histolytica* trophozoites cultured alone and those co-cultured with Caco-2 cells. Because the extracellular fragments were detected in culture supernatants but not in trophozoite lysates, fragment generation is likely to occur extracellularly or at the parasite surface. Furthermore, the effects of E-64 suggest the involvement of cysteine protease-dependent mechanisms.

There were at least three fragments observed in the culture supernatant, and the smallest fragment lacked the C-terminal region of the protein. Because the XEhI-20 antibody recognizes a conformational epitope of Igl1 rather than a specific linear amino acid sequence [11], the extracellular fragments identified in the present study may represent only a subset of all Igl1-derived fragments released into the culture supernatant. Additional fragments lacking the recognized conformational epitope may remain undetected. Our previous studies demonstrated that hemolytic and cytotoxic activities of Igl1 reside in the C-terminal region of the protein [8,10]. The presence of extracellular Igl1 fragments, particularly those retaining the C-terminal region, raises the possibility that Igl1 may function beyond the parasite surface. Consistent with this idea, it has been reported that Igl is not only localized at the site of trophozoite infection but also distributed in surrounding host tissues, suggesting that shedding and cleavage of Igl are naturally occurring phenomena [15]. Because of its cytotoxicity, Igl may therefore contribute to tissue damage beyond the immediate parasite surface. Indeed, we previously demonstrated that recombinant Igl1 fragments could attach to the surface of Caco-2 cells [8].

Igl1 is a cysteine-rich protein containing multiple CXXC motifs. *Giardia duodenalis* is known to secrete cysteine-rich surface proteins as virulence factors, contributing to cytotoxic effects on epithelial cells [16]. The parasite has at least 61 high-cysteine membrane proteins (HCMPs), which are secreted during trophozoite–epithelial interactions. Igl1 may similarly be secreted or shed upon contact with Caco-2 cells. However, the protein or mRNA expression levels of Igl1 did not differ between the single culture of *E. histolytica* trophozoites and the co-culture with Caco-2 cells.

Cysteine proteases are well-established virulence factors in protozoan parasites, contributing to host tissue degradation, immune evasion, and parasite survival [17,18]. In intestinal protozoa such as *Giardia duodenalis*, secreted cysteine proteases disrupt epithelial barrier integrity and degrade host defense molecules [19,20]. Similarly, in *Leishmania* spp. [21] and *Trypanosoma cruzi* [22], cysteine proteases play essential roles in host–parasite interactions and immune modulation. In this context, the protease-dependent processing of Igl1 observed in this study may represent an additional layer of regulation in host–parasite interactions.

The fragmentation of Igl1 was suppressed by the exogenous cysteine protease inhibitor E-64. Together with the fact that fragmentation was not observed in *E. histolytica* lysates, these findings suggest that Igl1 fragmentation likely occurs extracellularly or at the parasite surface. *E. histolytica* trophozoites express and secrete cysteine proteases, which are known virulence factors that not only directly damage host cells and tissues but also modulate host immune responses [23]. Our findings raise the possibility that these proteases not only act directly on host components but also regulate parasite-derived virulence factors such as Igl1 through proteolytic processing.

We also observed that the apparent molecular size of Igl1 differed between trophozoites cultured alone and those co-cultured with Caco-2 cells. Because these differences were already evident in intact Igl1 in cell lysates, they are unlikely to be explained solely by extracellular proteolytic processing. Rather, host-cell-associated conditions may influence the molecular state of Igl1 prior to cleavage. The underlying mechanisms remain unclear, as differences in transcript levels, protein folding, and N-terminal processing were not detected in this study.

To examine the contribution of soluble host-derived factors, we attempted to culture trophozoites in Caco-2-conditioned TYI-S-33 medium; however, trophozoites did not survive under these conditions, precluding further evaluation. Nevertheless, co-culture with Caco-2 cells consistently altered the molecular size of Igl1, suggesting that factors associated with direct host–parasite contact may underlie this effect.

Further studies to determine whether protease-dependent processing of Igl1 modulates its cytotoxic and adhesive activities may provide new insights into the regulation of parasite virulence. Whether this phenomenon can be generalized to other intestinal epithelial cell lines could be addressed by co-culturing *E. histolytica* trophozoites with HT-29, HCT116, and other relevant cell lines. In addition, identification of the protease(s) involved and determination of the cleavage site(s) will be important for understanding the mechanism underlying Igl1 fragmentation.

## Data Availability

Data is contained within the article or Supplementary Material.

## Supplementary Materials

The following supporting information can be downloaded at https://www.mdpi.com/article/10.3390/pathogens15060633/s1, Figure S1: Molecular species of Igl1 in the culture supernatant of *Entamoeba histolytica*.

## Abbreviations

The following abbreviations are used in this manuscript:

Gal	galactose
GalNAc	*N*-acetyl-D-galactosamine
kDa	kilodalton
PBS	phosphate-buffered saline
TBST	tris-buffered saline with 0.1% Tween-20
GPI	glycosylphosphatidylinositol
E-64	trans-Epoxysuccinyl-L-leucylamido-(4-guanidino)butane
MWCO	molecular weight cut off
BSA	bovine serum albumin
AP	alkaline phosphatase
CBB	Coomassie Brilliant Blue
