# Supplementary material for: Caco-2 Cell Co-Culture Alters the Molecular Size of Igl1 and Its Extracellular Fragments in Entamoeba histolytica
Source: Pathogens. 2026 Jun 15;15(6):633. doi: 10.3390/pathogens15060633 (PMC13304673; doi:10.3390/pathogens15060633)
Supplement: Supplementary file 1 [file pathogens-15-00633-s001.zip › Figure S1 legend.pdf]

Legends for Supplemental Figures

Figure S1. Molecular species of Igl1 in culture supernatant of *Entamoeba histolytica*. A: Three fragments of Igl1 (band numbers 1-3) were detected by western blot analysis with XEhI-20 antibody in culture supernatant (Sup). IgG derived proteins could be removed from the samples using protein G magnetic beads (- IgG). An arrowhead indicates recombinant protein G detached from the beads. Asterisks indicate the bands of IgG derived proteins. The samples were run in 4-12% Bis-Tris gels. B: Detached Caco-2 cells (black arrows) were confirmed as dead cells by a trypan blue exclusion assay and those cells clotted and floated in the culture medium. *E. histolytica* trophozoites were viable (white arrowheads).
