# Supplementary figures and images for: Caco-2 Cell Co-Culture Alters the Molecular Size of Igl1 and Its Extracellular Fragments in Entamoeba histolytica
Source: Pathogens. 2026 Jun 15;15(6):633. doi: 10.3390/pathogens15060633 (PMC13304673; doi:10.3390/pathogens15060633)

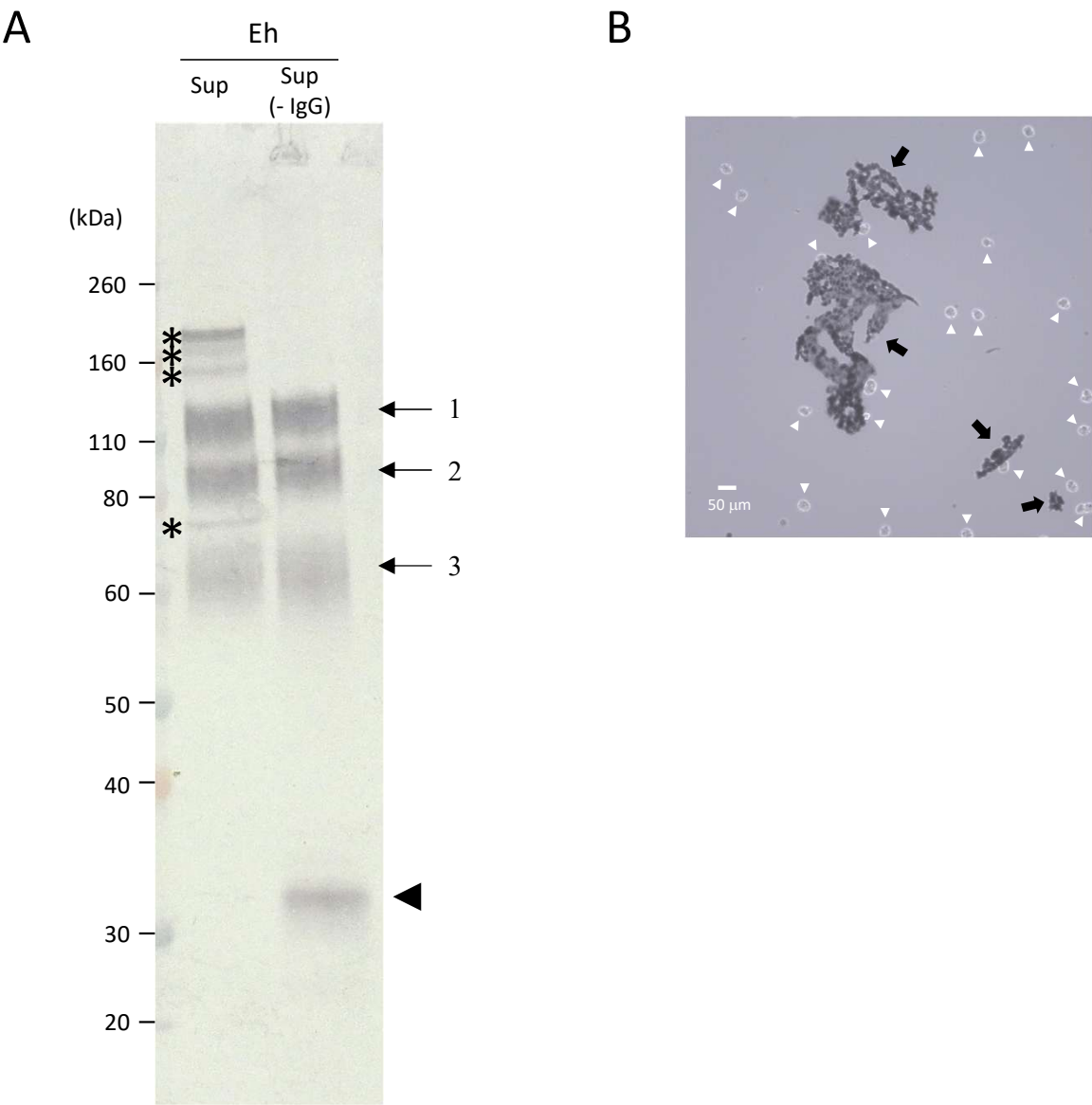

Supplement: Supplementary file 1 [file pathogens-15-00633-s001.zip › Supplemental Figure S1.pdf]
